# White Bread and Its Impurities

**Published:** 1911-04-15

**Authors:** 


### SPECIAL INSTITUTIONAL ARTICLE.

### WHITE BREAD AND ITS IMPURITIES.

### THE BLEACHING OF FLOUR IN RELATION TO PUBLIC HEALTH.

In his treatise on the "Adulteration of Food," published nearly a hundred years ago, Accum tells us, concerning bread, that "to produce the degree of whiteness rendered indispensable by the caprice of the consumers in London it is necessary (unless the very best flour is employed) that the dough should be bleached." The taste for white bread is therefore not one of recent cultivation. The method of making flour white, however, has changed, and instead of the alum which bakers of a former generation were accustomed to use for this purpose the millers of to-day employ processes of a highly technical nature embodying the results of more recent scientific discoveries. All commercial bleaching of flour at the present time depends on the use of nitrogen peroxide, which can be produced either by chemical or electrical means. By the chemical method the nitrogen peroxide is produced by the action of nitric acid on ferrous sulphate, the amount of gas formed being controlled by regulating the supply of acid and ferrous sulphate. A current of air charged with the gas is led through a revolving reel through which a stream of flour is continually flowing, and, being thoroughly exposed to the action of the gas, the flour is bleached when it emerges from the machine.

#### Bleaching by Electricity.

The electrical methods of bleaching depend upon the fact that an electrical sparking discharge in air results in the combination of a certain amount of the nitrogen and oxygen of air to form nitrogen peroxide; these methods are rapidly superseding chemical processes on account of their simplicity in operation and of the greater accuracy with which the degree of bleaching can be controlled and over-bleaching avoided. In the United States of America flour bleached by nitrogen peroxide is an adulterated product under the Food and Drugs Act, and such flour cannot legally be made, *except for export*. The fact that the sale in America of bleached flour is prohibited has frequently been used as an argument in favour of its prohibition in this country.

#### The Dangers of Bleaching Flour.

Other reasons have also been adduced and numerous representations on the subject made to the Local Government Board, in consequence of which instructions were given to Dr. J. M. Hamill to make inquiries as to how far the practice of bleaching obtains and to what extent, if at all, it is justified. Dr. Hamill's report (Cd. 5613) has just been issued, and it is of an interesting and very instructive character. Bleaching has not yet become universal in this country, even amongst the larger firms, for various reasons, and many millers have never bleached flour for sale. On the other hand, it is the custom of many millers when using a bleaching plant to bleach all the flour produced. In other cases the miller has found that his customers prefer high-grade flour unbleached, and has in consequence restricted bleaching to low grades only.

#### Millers' Profits from Bleaching.

As to the effect of bleaching on flour many contradictory statements are made, but, in reviewing them, Dr. Hamill comes to the conclusion that there can be no doubt that bleaching is capable of improving the colour and therefore the commercial value of the lower grades of flour; it also enables the miller to use cheaper wheats and to transform what would otherwise be a dark-coloured, cheap flour into a whiter, higher-priced article, the gain due to the enhanced price, which may amount to 6d. to 1s. a sack, going to the miller. There seems no sufficient evidence, however, that mere bleaching can counteract defects in flour due to unsoundness in the wheat from which it is milled. Since it is established that these processes are employed on a very considerable scale, the question arises whether any appreciable changes occur in flour as a result of bleaching by nitrogen peroxide.

#### Dr. Monier-Williams' Experiments.

As the conclusions of the different workers who have investigated this matter are contradictory, it was arranged that further experimental investigations should be made by Dr. Monier-Williams at the laboratory of the Local Government Board. The result of this work clearly shows that, with higher degrees of bleaching, definite changes are produced in the constitution of the flour. Among these are an increase in soluble proteins and carbohydrates; the oil of the flour acquires the character of an oxidised oil, and about six to seven per cent. of the nitrogen introduced into the flour as nitrogen oxide is absorbed by the oil. The analysis of a large number of samples of British-milled flour shows that none contained more than four parts per million of nitrate contents and most of them contained none at all or less than one part in two million. Dr. Hamill is of opinion, however, that it cannot be regarded as desirable that minute doses should be ingested day by day of a drug which in large single doses has marked action on the vascular system.

#### Harmful Effect of Bleaching on Digestion.

In order to make the inquiry more complete, Dr. Harden carried out certain experiments at the Lister Institute, and found that no obvious effects were produced on animals fed with highly bleached flour. Dr. Harden's report also contains an account of experiments in regard to peptic and pancreatic digestion of bleached and unbleached flour, and Dr. Monier-Williams gives corresponding results with salivary digestion. From these it appears that bleaching has a distinctly inhibitory effect on peptic digestion, but no observable effect on the pancreatic (proteolytic)

digestion of flour. Sodium nitrate was found to exert no inhibitory effect on the salivary digestion of starch, but in the case of starch treated with nitrogen peroxide, digestion was greatly retarded. Bleaching was also found to exercise an inhibitory effect on the salivary digestion of food. After reviewing the whole subject in the light of all these investigations, Dr. Hamill comes to the conclusion that the alterations in, and the additions to, flour which result from a high degree of bleaching cannot be regarded as free from risk to the consumer. The use of so-called flour "improvers," such as phosphoric acid and phosphates, which are said to increase the lightness and improve the quality and appearance of the loaf must, in Mr. Hamill's view, be regarded with considerable apprehension.

### Legislation Probable.

Dr. Hamill expresses the opinion, with which most people will agree, that it is not desirable that such an indispensable foodstuff as flour, the purity and wholesomeness of which are of first importance to the community, should be manipulated and treated with foreign substances, the utility of which, from the point of view of the consumer, is more than questionable. As a consequence of this report, steps will no doubt be taken at no distant date to give effect to the implied recommendations.

